# Exposure frequencies of single adverse childhood experiences and their association with psychological distress: evidence from a cohort study among emerging Swiss adults

**DOI:** 10.1186/s12889-025-25999-6

**Published:** 2025-12-24

**Authors:** Simon Marmet, Salome I.R. Bötschi, Neela Vetsch, Lina Stallmann, Jeannette Brodbeck

**Affiliations:** 1 School of Social Work, University of Applied Sciences and Arts Northwestern Switzerland, Riggenbachstrasse 16, Olten, 4600 Switzerland; 2 Swiss Center for Affective Science, University of Geneva, Geneva, Switzerland; 3 Department of Clinical Psychology, University of Bern, Bern, Switzerland

**Keywords:** Adverse childhood experiences, Emerging adulthood, Prevalence, Psychological distress, Peer maltreatment, Single adversity approach

## Abstract

**Background:**

Adverse childhood experiences (ACE) are significant transdiagnostic risk factors for impaired psychosocial functioning throughout life. Most research on ACE has relied on composite scores, limiting conclusions about specific adverse experiences. The aim of the present study was to describe in detail the exposure frequencies of a wide range of single adverse experiences (SACEs) and investigate their association with psychological distress among emerging adults at the individual and the population levels.

**Methods:**

A total of 2528 emerging Swiss adults aged 18 to 22 years from a random population sample participated in the first wave of a cohort study. ACE measures included 39 specific experiences covering neglect (emotional, physical), abuse (emotional, physical, sexual), witnessing violence, peer maltreatment and parental psychopathology. ANOVA models estimated bivariate associations between SACE exposure frequencies and psychological distress.

**Results:**

The most frequently reported child maltreatment subtypes were emotional neglect and abuse (38.4% resp. 30.8%). Regarding SACEs, more than 75% reported “witnessing the parents arguing fiercely” and verbal maltreatment by peers. SACEs were strongly associated with psychological distress in emerging adulthood, though the strength varied by type and frequency of exposure. Physical and sexual abuse experiences were strongly associated with psychological distress even when experienced only rarely. In contrast, SACEs in the domains of emotional abuse, emotional neglect and verbal abuse by peers correlated with psychological distress primarily at higher exposure frequencies. At the population level, frequently occurring SACEs, such as verbal abuse by family, explained the largest proportion of variance in psychological distress.

**Conclusions:**

The impact of adverse childhood experiences on psychological distress among emerging adults depends on the type of SACE and their exposure frequency. These findings highlight the importance of analyzing single adversities and their frequencies alongside dimensional and pattern-based approaches in ACE research.

**Trial registration:**

NCT05122988; November 17, 2021.

**Supplementary Information:**

The online version contains supplementary material available at 10.1186/s12889-025-25999-6.

## Highlights

- Most research on adverse childhood experiences (ACE) relies on composite scores or dimensional approaches. This study examines the detailed prevalence of exposure frequencies for specific adverse experiences (ranging from “never” to “very often”) and their association with psychological distress in emerging adults.
- The impact of ACE on psychological distress varies greatly depending on the specific adverse experience and its exposure frequency. Type 1 SACEs (e.g. physical or sexual abuse) show a strong association with psychological distress even at low exposure frequencies. Type 2 SACEs (e.g. emotional neglect or witnessing parents fighting) were reported by a large proportion of participants and show weaker associations with psychological distress at low frequency levels. However, these associations increase at higher exposure levels.
- At the individual level, the highest risk of psychological distress is linked to exposure to Type 1 SACEs. Conversely, at the population level, Type 2 SACEs have the most significant impact on psychological distress. While their individual effect is less severe, their prevalence is sufficient to influence the mental health of a substantial portion of the population.

**Supplementary Information:**

The online version contains supplementary material available at 10.1186/s12889-025-25999-6.

## Introduction

Approximately 30 to 60% of children worldwide experience adverse childhood experiences (ACE) [1–3]. ACE is an umbrella term for distinct experiences that occur before the age of 18 and pose risks to physical, emotional and social well-being across the life course. ACE encompass emotional, physical and sexual abuse, emotional and physical neglect, exposure to intimate partner violence or other household dysfunctions [4, 5] as well as peer maltreatment [6–8].

ACE are a well-established transdiagnostic risk factor for impaired psychosocial functioning [8–11] and a wide range of mental disorders such as depression and anxiety, and negative physical health outcomes throughout life [12–14]. Recent research underscores the significant impact of peer maltreatment and bullying during childhood and adolescence, which are linked to reduced physical, emotional, and social well-being.

A common finding in the literature is a dose-response relationship between a higher number or higher score of ACE and negative psychosocial outcomes [12, 15, 16]. Regarding specific dimensions of ACE, some studies identified emotional abuse as the strongest predictor of depression and anxiety disorders, followed by neglect and physical abuse [17–19]. In contrast, Daníelsdóttir et al. [12] found that physical neglect and sexual abuse exhibited the strongest associations with any adult psychiatric disorder.

### Composite score and single-adversity-approaches to investigate prevalence and consequences of ACE

Most ACE questionnaires assess the exposure frequencies (for example from “never” to “very often”) to a number of single adverse childhood experiences (SACEs) on various dimensions of ACE, e.g. the frequency of being called names by parents as experience of emotional abuse or the frequency of being punished with hard objects as experience of physical abuse. Frequently, SACEs are aggregated into broader measures, such as the total number of ACE subtypes, composite scores, or dimensions or patterns of ACE, to explore their associations with psychosocial outcomes [20]. Aggregating SACEs into composite scores is particularly valuable for complex models investigating the mechanisms linking ACE to psychosocial outcomes. Such models often require the consolidation of individual ACE to maintain feasibility and interpretability while capturing the overarching impact of ACE exposure.

However, this approach has several limitations and sacrifices detailed insights into the impact of specific SACEs [20]. The composite score approach does not account for the diversity of experiences encompassed by the umbrella term ACE, which spans emotional, physical, and sexual domains. Also, within one domain, experiences can vary considerably. For example, the severity of physical SACEs varies widely, ranging from a slap to serious physical injuries requiring medical treatment, and their impact may differ accordingly. Thus, using an ACE composite score implicitly assumes that all SACEs have an equal impact on outcomes, which oversimplifies their distinct effects [20, 21].

A complementary approach is the single-adversity-approach [20], which focuses on the association between specific SACEs or dimension of SACEs and psychosocial outcomes. Still, SACEs are often assessed as binary variables (present or absent) or as a singular score on a specific SACE dimension which overlooks critical questions about thresholds, i.e., the exposure frequency at or above which distinct ACE are significantly associated with long-term psychological distress.

For instance, a singular occurrence of severe physical or sexual violence can have severe and lasting consequences on wellbeing warranting classification as a severe event even if it occurs only once [22]. By contrast, experiences such as being called names by family members or feeling unimportant may have minimal consequences when rare. However, if such SACEs occur frequently, they can also lead to significant psychological distress, both in childhood and adulthood. Additionally, the impact on an individual will also depend on the specific circumstances and personal factors such as age, personality, resilience, coping strategies, and the availability of social support [23].

Although exposure frequencies of SACEs are often incorporated into composite scores, these methods do not allow for the differentiation of their unique impacts across SACEs. Similarly, single-risk approaches using binary or dimensional assessments of ACE fail to capture the nuanced effects of varying exposure frequencies. To the best of our knowledge, no study to date has examined the specific associations between exposure frequency thresholds of a broad range of SACEs and psychological distress in emerging adulthood.

The present study addresses this gap by integrating exposure frequencies into the single-adversity approach. This enables the investigation of exposure frequency thresholds across a diverse range of SACEs and their associations with psychological distress in emerging adulthood. Furthermore, it examines these associations at the individual as well as at the population level.

### The present study

This study provides a comprehensive descriptive overview of the prevalence of a broad range of specific adverse childhood experiences (SACEs) and examines their associations with psychological distress at both ind+

The first aim was to investigate the detailed prevalence rates and exposure frequencies of a wide range of SACEs. Participants reported how often they experienced specific forms of adversity, including parental maltreatment, peer maltreatment, witnessing domestic violence, and parental psychopathology, on a scale from “never” to “very often.” These self-reported prevalence rates from a general population sample complement existing data sources, such as medical and social service records, and can inform the development of prevention and intervention strategies [22].

The second aim was to analyze how psychological distress in emerging adults varies according to how often individuals experienced a specific SACE. The third aim was to estimate, at the population level, the proportion of variance in psychological distress attributable to the exposure frequencies of each SACE. To achieve this, we integrated both the prevalence of each exposure frequency and its association with psychological distress among those affected.

## Methods

### Sample

The Swiss Federal Office of Statistics provided mailing addresses for a random sample of emerging adults aged 18 to 21 years residing in private households in German-speaking municipalities in Switzerland (see Brodbeck et al. [24]). A total of 15,181 individuals received the study information and were invited by postal mail to complete an online questionnaire hosted on the REDCap platform [25, 26]. Due to a lower-than-expected response rate, and to ensure sufficient sample size for the intervention component of the FACE project [27], an additional 167 participants were recruited through universities, teacher training colleges, and military training schools.

In total, 2,439 participants from the random sample and 167 from the additional recruitment pool provided electronic informed consent, and 2,538 started the questionnaire. Ten participants were excluded due to implausible response patterns, such as line patterns (8 cases) or a completion time below 10 min (2 cases). The analytic sample for this study consists of 1,886 participants who completed the ACE questionnaires (Table 1). Compared with the total invited random sample (*n* = 15,181), the main analytical sample (*n* = 1,759) was approximately one month younger (*p* < .001), more likely to be female (67.8% vs. 50.2%; *p* < .001), and more likely to be of Swiss nationality (89.1% vs. 81.4%; *p* < .001). The additional recruitment sample (*n* = 127) was, compared with the main analytical sample, approximately one year older (*p* < .001), more likely to be female (*p* < .001), and non-significantly more likely to be of Swiss nationality (*p* = .137).

**Table 1:** Descriptive statistics

	mean / *n*	SD /%
Analytic sample	1886	
Age (mean / SD)	19.39	1.34
Gender		
male	587	31.2%
female	1257	66.9%
other	35	1.4%
Relationship status		
single	1191	63.6%
in a relationship	657	35.1%
married	23	1.2%
divorced/separated	1	0.1%
Living situation		
living alone	72	3.8%
with parents	1537	81.7%
with partner	45	2.4%
with friends/colocation	163	8.7%
other	64	3.4%
Professional situation		
vocational training	434	23.1%
interim year	80	4.3%
school (Gymnasium, other)	440	23.4%
university	554	29.5%
employed	330	17.6%
unemployed	40	2.1%
Nationality		
Swiss	1290	68.5%
Swiss with migration background	402	21.3%
Non-Swiss nationality	191	10.1%
Psychological distress (BSI-18)	13.85	11.6

### Measures

#### Adverse childhood experiences

*Child maltreatment* was assessed with the widely used Childhood Trauma Questionnaire (CTQ; [28, 29] which includes subscales for physical and emotional neglect, physical and emotional abuse, and sexual abuse. Each subscale contains five SACE with the response categories for exposure frequencies 1 = “never”, 2 = “rarely”, 3 = “sometimes”, 4 = “often” and 5 = “very often”, referring to experiences up to the age of 18 years. Reverse coded items were recoded so that for each item a score of 1 represents the most positive outcome and 5 the most negative. Subscale scores were calculated by summing the item responses, and severity was categorized into “none to minimal,” “slight to moderate,” “moderate to severe,” and “severe to extreme” based on thresholds established by Häuser et al. [29].

Witnessing domestic violence (5 items) and experiences of verbal, physical, and sexual abuse by peers (6 items) were assessed using items adapted from the German version of the Maltreatment and Abuse Chronology of Exposure (MACE) scale [30, 31]. Response categories were aligned with the CTQ questionnaire for consistency.

Parental and sibling psychological and substance use problems were assessed using items adapted from the C-SURF study [32] Seven items captured whether participants’ mothers, fathers, experienced substance use problems, psychological issues, or aggressive behavior. Responses for parents were summarized and coded as follows: no parent affected (0), one parent affected (1), and both parents affected by psychopathology (2).

#### Psychological distress

Psychological distress was measured using the 18-item version of the Brief Symptom Inventory (BSI-18) [33]. This widely used self-report screening tool is suitable for assessing overall psychological distress in general populations [34]. It comprises three subscales — depression, anxiety, and somatization — each subscale containing six items rated on a 5-point Likert-scale (0 to 4). The total BSI-18 score has a range from 0 to 72. Intercorrelations between subscales ranged from 0.53 to 0.68, and the total BSI-18 scale demonstrated high internal consistency (Cronbach’s alpha = 0.91). Given the high internal consistency of the BSI-18 and to reduce complexity of the analysis, only the BSI-18 total score was used for analysis.

#### Sociodemographic variables

Sociodemographic variables included age, gender, living situation, professional situation, relationship status and nationality.

### Statistical analysis

All statistical analyses were performed using SPSS 27.0. Data was collected online and rigorously reviewed for inconsistencies, suspicious response patterns, and implausible values. Inconsistent or highly implausible extreme values were coded as missing. For the CTQ subscales, if no more than two items were missing, the sum score was calculated based on the average of the remaining items and rescaled to the full scale. Descriptive statistics for exposure frequencies of each SACE were calculated using all available cases, with pairwise deletion applied to all analyses. Gender differences (reference group: women) in the distribution of exposure frequencies for SACEs were assessed using chi-square tests, while differences in ACE scale means were tested with bootstrapped t-tests.

ANOVA analyses were conducted to evaluate the relationship between psychological distress (z-standardized, with mean = 0 and SD = 1) as the outcome and each SACE as categorical predictors, using the lowest frequency level (“never”) as the reference group. Analysis was adjusted for gender (reference: women) and age (continuous). Psychological distress was z-standardized for easier interpretation of the effect sizes: As z-standardized mean differences correspond to Cohen’s d, thresholds for interpreting marginal means were adapted from Cohen’s guidelines (Cohen, 1977): 0.5 (medium effect), 0.8 (large effect), and an additional threshold of 1.2 (very large effect). Marginal means for the non-standardized BSI-18 score are reported in Supplementary Table S4. Marginal means were reported instead of mean differences relative to the reference group to facilitate comparison of effect sizes across different SACEs. These marginal means provide an estimate of the association between each SACE and psychological distress among individuals reporting specific exposure frequencies (second aim).

To address the third aim, the total sample variance in psychological distress explained by each SACE was estimated using partial eta squared within the same ANOVA analyses. Partial eta squared accounts for the interaction of effect size and prevalence, yielding a large value when a SACE has both a substantial effect and a high prevalence. Moderate values occur when the effect is small but the prevalence is high or vice versa. While partial eta squared is an unbiased measure of the sample effect, it tends to overestimate the population effect. However, this bias diminishes with larger sample sizes [35]. Effect sizes for partial eta squared are reported as percentage of variance explained and interpreted comparatively across different exposure frequencies and different SACEs rather than against specific thresholds [36]. Given our study’s substantial sample size, we consider partial eta squared a reliable estimate of population-level effects.

To account for the large sample size, which can make even minor effects statistically significant, and to address potential inflation of significant results due to multiple comparisons, we focused on the absolute effect sizes of marginal means and partial eta squared rather than relying on p-values for interpretation.

## Results

### Prevalence of SACEs

**Fig. 1:**
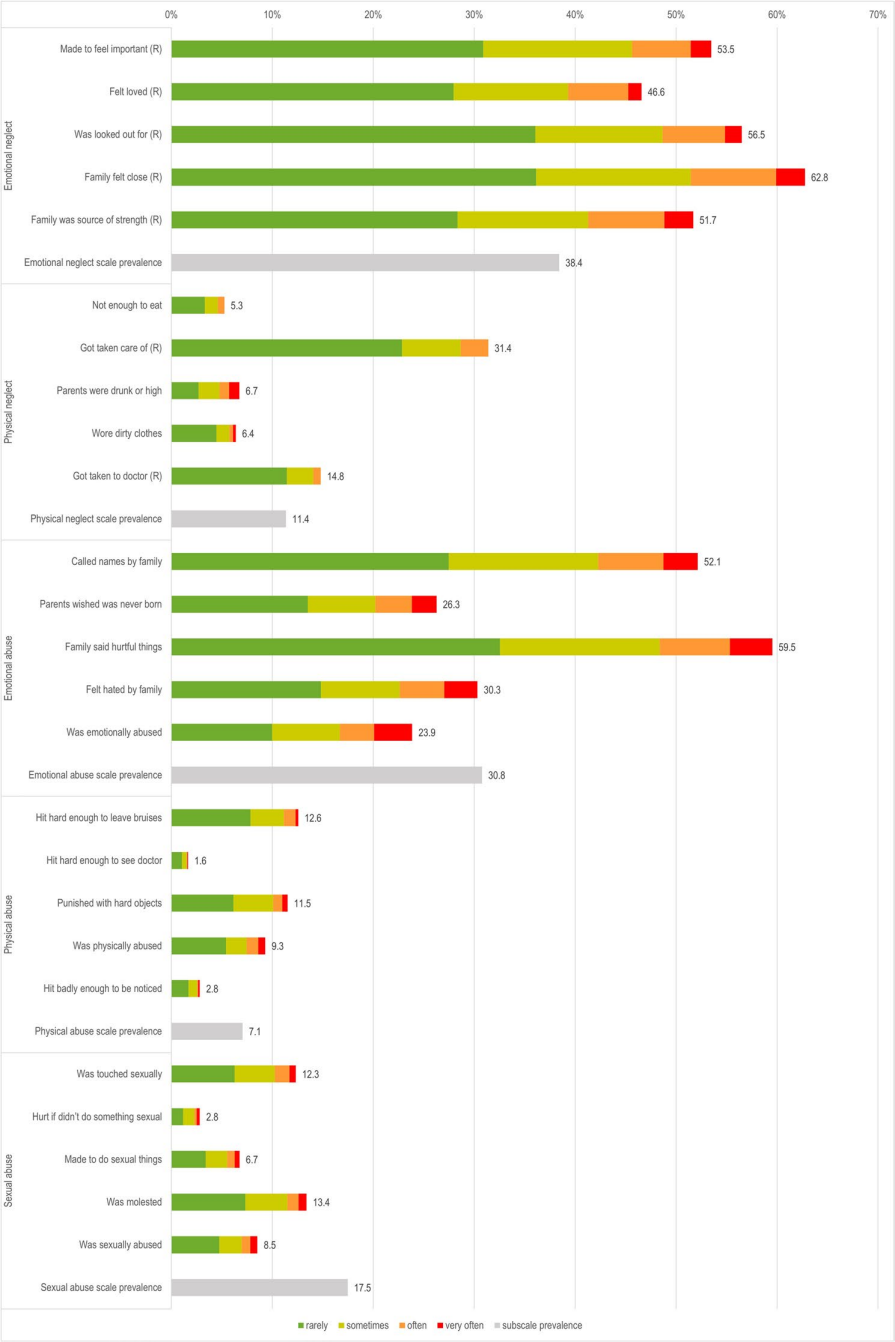
Caption. Prevalence of exposure frequencies of adverse childhood experiences from the CTQ questionnaire. Alt Text: A stacked bar chart showing the prevalence of each exposure frequency from rarely to very often for each of the 25 SACE of the CTQ questionnaire. The chart also displays the overall prevalence for each of the five CTQ subscales. The most frequently reported SACEs fall under the Emotional Neglect and Emotional Abuse subscales. Note: Only total prevalence rates for each SACE and ACE subscale are reported. Prevalence rates for exposure frequencies of each SACE by gender are available in Supplementary Table S1, and for scales in Supplementary Table S2

**Fig. 2:**
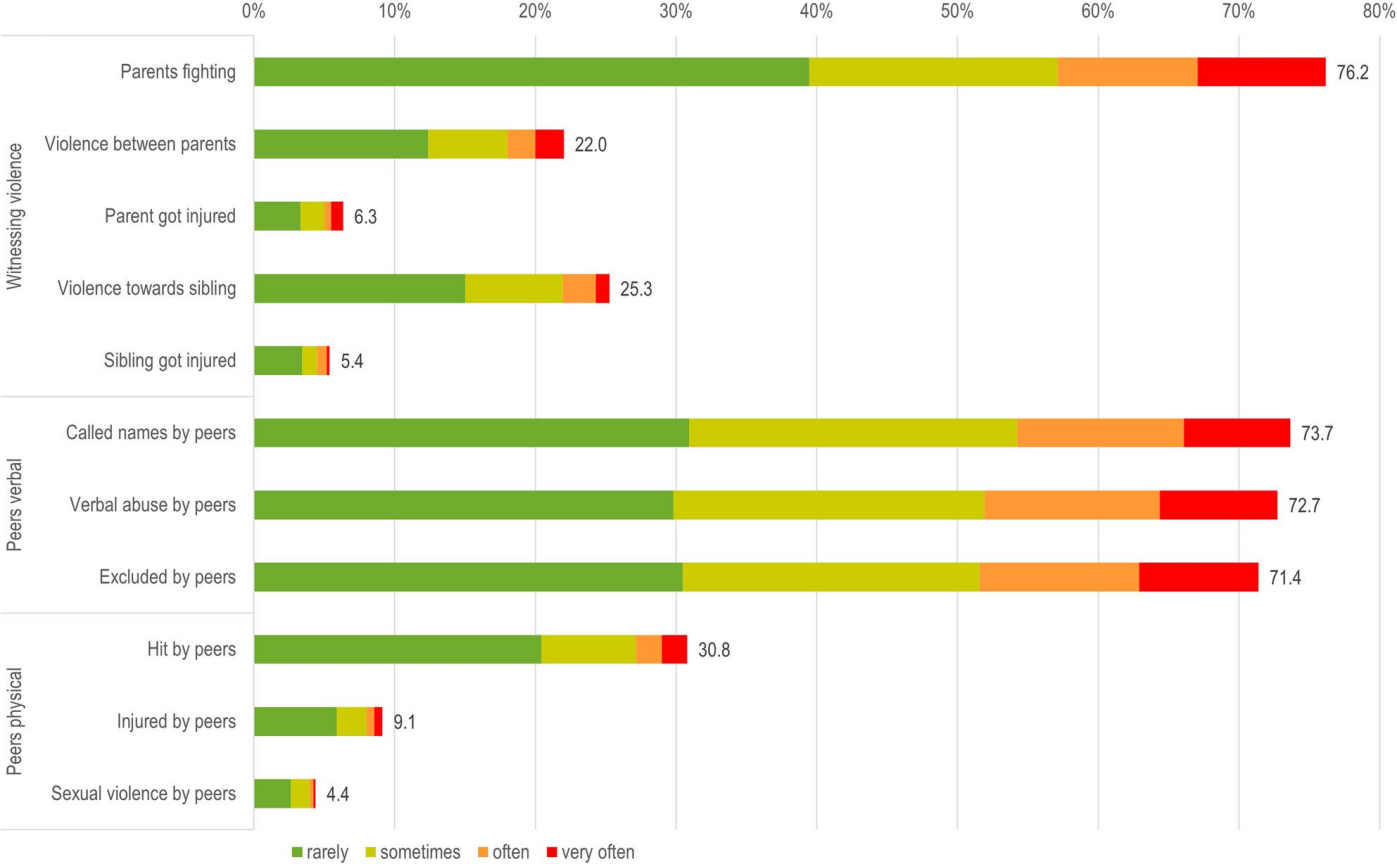
Caption. Prevalence of exposure frequencies of witnessing violence and peer maltreatment from the MACE questionnaire. Alt Text: A stacked bar chart showing the prevalence of each exposure frequency, from rarely to very often for each of the 11 SACE in the two MACE subscales. The most frequently reported SACEs fall under the subscale of verbal abuse by peers and witnessing parents fighting with each other. Note: Only total prevalence rates for each SACE and ACE subscales are reported. Prevalence rates for exposure frequencies by gender are available in Supplementary Table S1. Prevalence rates for MACE subscales are not reported due to the absence of validated cut-offs

Using the thresholds defined by Häuser et al. [29] for the CTQ subscales, the most frequent reported subtypes of child maltreatment were emotional neglect (EN, 38.4%) and emotional abuse (EA, 30.8%). Physical neglect was reported by 11.4% of the sample, physical abuse (PA) 7.1% and sexual abuse (SA) 17.5%, see Figs. 1 and 2. Witnessing violence (WV) and peer maltreatment (PM) were assessed with the MACE, which does not provide validated cut-offs. Thus, scale-level prevalences for these two categories are not reported.

Detailed prevalences for each exposure frequency of all SACEs are provided in Figs. 1 and 2. In summary, more than 75% of the participants reported “witnessing the parents arguing fiercely” or verbal maltreatment by peers (“being called names”, “verbal abuse by peers” and “excluded by peers”) at least rarely. Then, 48%-62% of the participants reported SACEs on the emotional neglect dimension of the CTQ. Two other frequently reported SACEs were the emotional abuse SACEs “called names by family” (52.1%) and “family said hurtful things” (59.5%). In contrast, less frequent SACEs on the emotional abuse dimension were parents wishing the participant had never been born (26.3%), feeling hated by family (30.3%), and being emotionally abused (23.9%). Thus, more than half of participants experienced family conflict and verbal maltreatment by peers.

Overall, SACEs in the physical domain were less frequently reported than emotional SACEs. Around 10% of the participants reported SACEs in the domain of physical abuse by adults, e.g., “being hit hard enough to leave bruises”, were “punished with hard objects” or indicated being “physically abused”. Nevertheless, 2.8% of the participants reported being “hit badly enough to be noticed” and 1.6% reported being “hit hard enough to see a doctor”, which was the least frequently reported SACE. Regarding sexual abuse by adults, 12.3% of the participants reported “was touched sexually”, 13.4% “was molested”, and 2.8% “hurt if I didn’t do something sexual”.

Verbal maltreatment by peers was prevalent, with 73.7% reported “called names by peers”. In the category of physical peer maltreatment, 30.8% reported “hit by peers”, while fewer reported being injured (9.1%) or experiencing sexual violence by peers (4.4%).

Prevalence rates for psychological or substance use problems among family members are presented in Table 2. A total of 17.1% of the participants reported problems of the mother, 15.9% of the father and 6.6% of both parents. The most frequently reported problems included depression of family members (34.5%) followed by other psychological problems (25.4%) and aggressive behavior (23%).

**Table 2:** Parental psychopathology

Type of problem	father	mother	both parents
Substance use problems			
Alcohol problem	5.8%	1.8%	1.0%
Drug problem	1.8%	0.4%	0.1%
Psychological problems			
Depression	5.5%	14.0%	2.9%
Delusions/hallucinations	0.5%	1.3%	0.0%
Suicide attempt	0.7%	1.6%	0.1%
Other psychological problems	5.0%	8.2%	2.7%
Aggressive behavior problems			
Aggressive behavior	10.0%	2.7%	2.2%
Any problem	15.9%	17.1%	6.8%

Note: Prevalence rates by gender are reported in Supplementary Table S3

Gender differences: Women reported significantly higher mean scores for emotional abuse, sexual abuse, witnessing violence, and verbal peer abuse (Supplementary Tables 1 and 2). Significant differences in frequency distributions were observed for 18 out of 39 SACEs. Notably, 35 participants identifying as non-binary/other reported significantly higher levels across nearly all ACE subtypes, except for physical abuse.

Prevalences for each exposure frequency of SACEs, disaggregated by gender, are detailed in Supplementary Tables 1 and 3. Severity levels and scale means are provided in Supplementary Table 2.

### Marginal means of psychological distress by SACE exposure frequencies

Table 3 presents the associations between exposure frequency for each SACE and psychological distress, as measured by z-standardized marginal means. Marginal means for the non-standardized BSI-18 score are reported in Supplementary Table S4. Generally, higher exposure frequencies (e.g., “often” or “very often”) were associated with higher levels of psychological distress. For example, the SACE “parents wished I was never born” was in the range of a medium effect size when reported “very rarely” (0.58), showed a large effect size when reported “sometimes” (0.92), a very large effect size when reported “often” (1.34) and when reported “very often” (1.92). However, these severe SACEs were reported infrequently at higher exposure levels, requiring cautious interpretation of effect sizes.

**Table 3:**
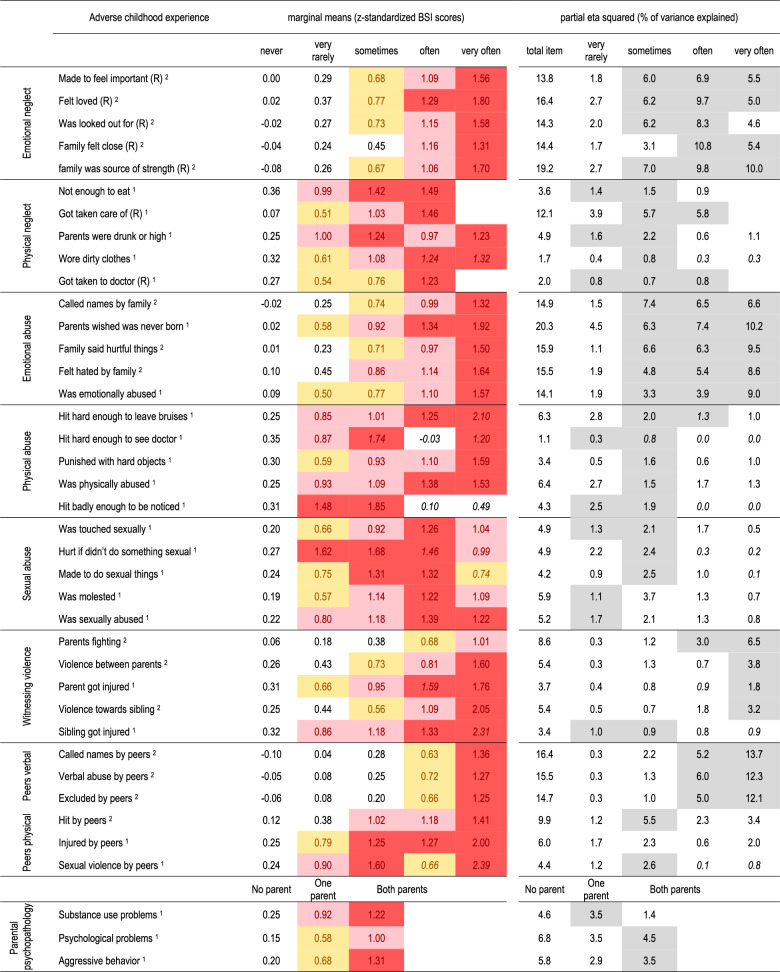
Associations between specific sace’s exposure frequencies and psychological distress (BSI score), adjusted for age and gender

Coefficients in italic are based on cells with a prevalence of less than 0.5% and should be interpreted with caution. Yellow: at least medium effect (≥0.5), light red: at least large effect (≥0.8), dark red: very large effect (≥1.2). Partial eta squared is reported for each exposure frequency level (reference “never”), and for the total of the item across all subtypes. Light grey cells for eta squared are cells with highest contribution within the line. All statistics are independent of each other and adjusted for age and gender. ^1^: Type 1 item: effect already at low frequency ^2^: Type 2 item: no or only small effect at low frequency. Classification into item Types is for illustration purposes only and approximate

Physical or sexual abuse experiences, although less frequently reported overall, showed strong associations (Cohen’s d ≥ 0.8) even at lower exposure frequencies. For instance, “was sexually abused” and “experienced sexual violence by peers” were strongly associated with psychological distress at a frequency of “sometimes.” Notably, “hit badly enough to be noticed” and “hurt if I didn’t do something sexual” showed large associations (1.48 and 1.62, respectively) even when reported “rarely.”

**Fig. 3:**
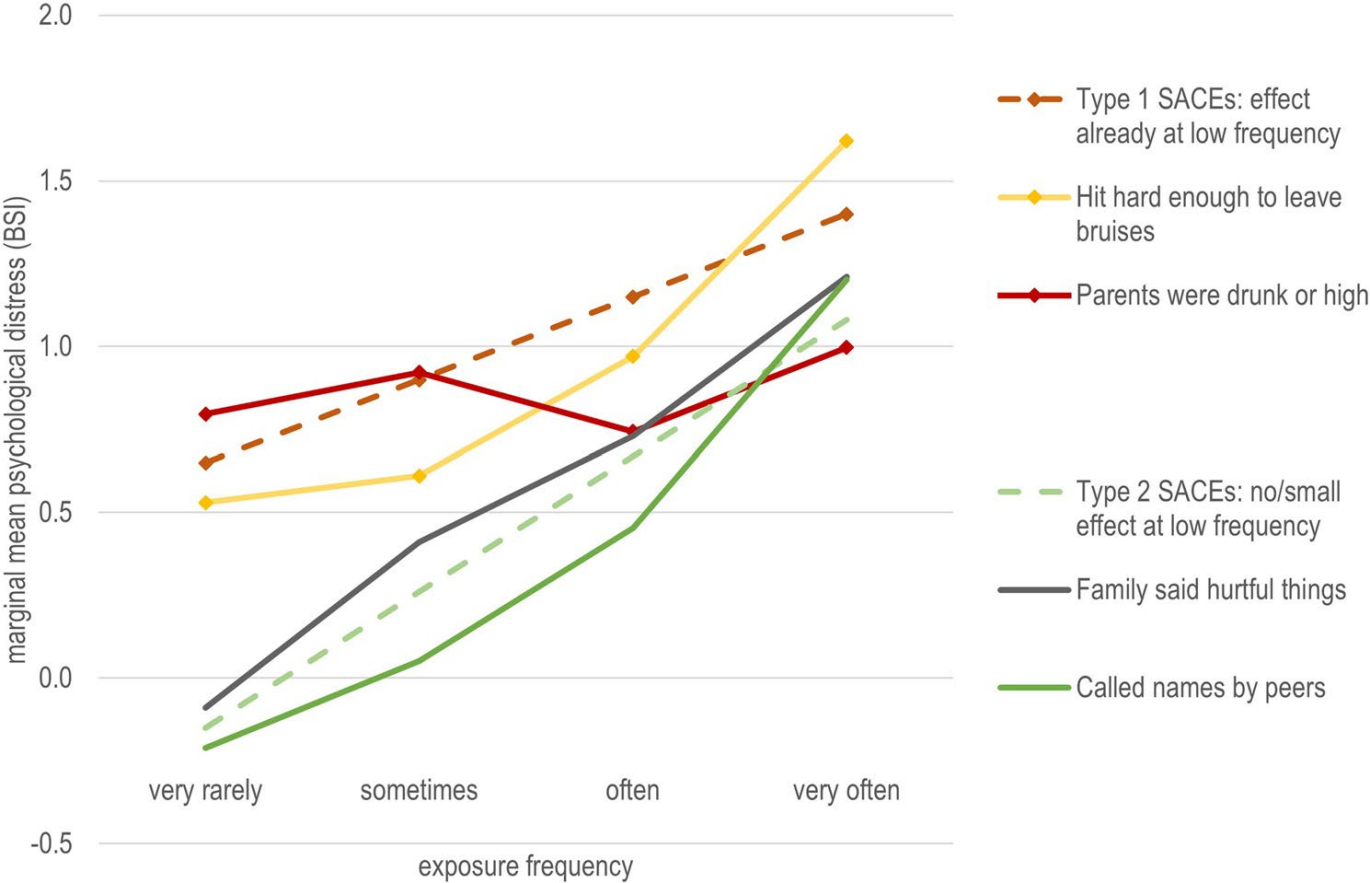
Caption: Prototypical SACEs and their association with psychological distress across exposure frequencies. Alt Text: A line chart illustrating marginal means for psychological distress across exposure frequencies of two types of SACEs. Type 1 SACE shows an effect on psychological distress even at low exposure frequencies. Type 2 SACE shows little or no effect at low exposure frequencies.

For illustrative purposes, Fig. 3 presents two types of SACE, each represented by two prototypical examples.

Figure 3 illustrates two patterns of SACEs based on their association with psychological distress as prototypical examples:

- Type 1 SACEs: These had strong associations with psychological distress even at low exposure frequencies, with steady or increasing associations with increasing exposure frequency. These SACEs, often less frequently reported, were primarily in the domains of physical neglect (e.g., "not enough to eat"), physical and sexual abuse (both familial and peer-related), and witnessing violence involving physical aggression.
- Type 2 SACEs: These showed low associations with psychological distress at low exposure frequencies but an increasing (or consistently high) association at higher exposure frequencies. These SACEs were reported by a large proportion of participants at low exposure frequencies and included mainly emotional neglect (EN), certain emotional abuse (EA) experiences, witnessing parents fighting, and verbal peer abuse. While the marginal means for "very rarely" exposure levels were higher than the reference group ("never"), they were typically close to the sample average (0).

### Variance in psychological distress explained by exposure frequencies of SACEs

Table 3 also reports partial eta squared, providing an estimate of the variance in psychological distress explained by each SACE’s exposure frequencies at the population level.

Type 1 SACEs had high partial eta squared values even at low exposure frequencies, with either stable (e.g., “was physically abused”) or decreasing (e.g., “hit hard enough to leave bruises”) values at higher frequencies. Despite strong individual-level associations at higher frequencies, their low prevalence meant they contributed minimally to overall variance in psychological distress.

Type 2 SACEs were reported by a large proportion of participants at low frequencies, resulting in low partial eta squared values at these levels due to weaker associations with psychological distress. However, at higher exposure frequencies, which were reported by fewer participants, their associations with psychological distress were much stronger. This led to relatively higher partial eta squared values at medium-to-high exposure frequencies. Overall, Type 2 SACEs, primarily in the domains of emotional abuse (EA), emotional neglect (EN), and peer verbal abuse, accounted for the largest proportion of explained variance in psychological distress across all exposure frequencies.

## Discussion

This study utilized a large random sample of Swiss emerging adults to examine the prevalence of various single adverse childhood experiences (SACEs) and the extent to which the frequency of exposure to SACEs is associated with psychological distress at both individual and population levels.

More than half of participants reported having experienced some form of child maltreatment (i.e. emotional abuse or neglect, physical abuse or neglect or sexual abuse) as defined by Häuser et al. [29] for the CTQ subscales. Emotional neglect (38.4%) and emotional abuse (30.8%) were the most common subtypes, whereas physical neglect (11.4%) and physical abuse (7.1%) were less frequent. Sexual abuse was reported by 17.5% of participants, potentially reflecting the larger proportion of women in our sample, who are at greater risk for SA [37, 38]. Compared to Swiss data based on reports of child protection services [37], the prevalence of emotional abuse and emotional neglect was notably higher, suggesting underreporting of emotional maltreatment cases in child protection services compared to physical maltreatment. International comparisons show slightly higher rates of any subtype of ACE, i.e. 57.7% [3], 62.8% in an US sample [1] and 62.2% in an Australian sample [2]. Regarding subtypes of ACE, a US study found similar rates for emotional abuse (34.5%) and sexual abuse (14.1%), but higher prevalences of physical abuse (25.2%) [1]. In an Australian study, the prevalence of physical abuse (32.0%) and sexual abuse (28.5%) were considerably higher than in our study [2]. The review of meta-analysis around the globe by Stoltenberg et al. [5] found lower self-reported emotional neglect prevalence, but higher physical abuse rates.

Witnessing violence and peer maltreatment were also frequently reported, though no established cut-offs for comparing prevalences of witnessing violence and peer maltreatment exist for these categories in the MACE. Nonetheless, a substantial portion of participants experienced SACEs related to verbal abuse by peers or witnessing violence, while physical peer maltreatment was less common.

Notably, participants identifying as non-binary reported significantly higher levels of SACEs compared to participants identifying as women or men. This is consistent with a recent systematic review [39]. Gender nonconform behaviors and the rejection of one’s gender identity may increase the risk for family maltreatment and peer victimization. Similarly, parental rejection of one’s sexual orientation was found to be related to psychological distress later in life and internalized homophobia and social support mediated the association between parental rejection and psychological distress [40]. These findings suggest that ACE may aggravate the compounded challenges that non-binary people face in society and aligns with recommendations to consider family dynamics when supporting non-binary individuals [39, 41].

### Marginal means of psychological distress by SACE exposure frequencies

Our findings showed that individuals with higher exposure frequencies of SACEs reported, on average, higher psychological distress. However, the threshold at which an association with psychological distress emerged, and the extent to which psychological distress increased across exposure levels, varied considerably across different SACEs.

For illustration, we discuss these results for two broad types of SACEs. Type 1 SACEs include experiences of physical and sexual abuse, which were reported by fewer participants but showed strong associations with psychological distress even at low exposure frequencies. Type 2 SACEs typically encompass experiences of EN, EA, witnessing parents arguing fiercely and verbal abuse by peers and were reported by over half of our participants, predominantly at a low exposure frequency (“rarely” or “sometimes”). Type 2 SACEs showed weak or no association with distress at low exposure levels (“rarely” or “sometimes”). However, at higher exposure frequencies (“often” or “very often”), their association with psychological distress became as strong as that of Type 1 SACEs.

These findings are consistent with reflections from Stoltenborgh et al. [5] that abuse, particularly SA and PA (type 1 SACEs in our study), may have a profound impact even from a singular event. In contrast, emotional abuse and neglect types of maltreatments (type 2 SACEs in our study) require chronic exposure to significantly impact psychological distress.

Our findings regarding type 2 SACEs merit further discussion. At low exposure levels, some Type 2 SACEs may fall within the range of normal developmental challenges and even provide opportunities for developing coping skills for social stress situations. This perspective recognizes that challenges and stress are to some degree inherent in the process of growing up, and in family and peer relationships. Nevertheless, when chronic or severe, these experiences cross a threshold, becoming profoundly harmful. ACE can be defined as experiences that “require significant adaptation by the developing child in terms of psychological, social and neurodevelopmental systems, and which are outside of the normal expected environment” [42]. However, what can be considered outside of the normal expected environment may not only depend on the characteristic of an adverse experience but also on how frequently a person was exposed to them [5, 22].

In line with previous research [8–11], our results highlight the importance of peer maltreatment, including verbal, physical, and sexual abuse, regarding psychological distress. Physical and sexual peer abuse had comparable associations to family-based physical abuse. For verbal abuse by peers, as in the family environment, exposure frequencies are important to consider. Verbal abuse by peers, while less impactful at low exposure levels, demonstrated significant associations with distress at higher frequencies. This underscores the importance of including peer maltreatment in ACE frameworks, as its associations with psychological distress are of similar strength to maltreatment within the family environment [6, 7, 43].

Consistent with previous research [15], parental psychopathology was a risk factor for psychological distress during emerging adulthood. However, we found that having one parent with psychopathology was only moderately associated with psychological distress. This suggests that the impact of one’s parent’s psychopathology on the offspring may be mitigated by the presence of a parent without psychopathology [44].

### Population-level variance in psychological distress explained by SACEs

In addition to estimating marginal means across groups defined by SACE exposure frequency, we also examined the extent to which SACEs contributed to psychological distress at the population level. For this purpose, we used partial eta squared as a measure of explained variance. The observed variance explained reflects both the strength of the association with distress and the prevalence of exposure: SACEs with strong associations at the individual level may nevertheless explain little variance at the population level if they are infrequently reported. Intriguingly, our results show that the contribution from type 2 SACEs (e.g. emotional neglect) was higher than the contribution of the more severe type 1 SACEs (e.g. severe physical abuse). Despite strong individual-level associations with psychological distress, type 1 SACEs were less prevalent and therefore had a smaller population-level impact. In contrast, due to their higher prevalence, Type 2 SACEs (e.g., EN, EA, and peer verbal abuse) accounted for a greater portion of the variance in psychological distress across the population, even though their associations with psychological distress were weaker at lower exposure levels.

Overall, the greatest contribution to psychological distress often does not come from the most severe or the most frequently reported exposures. Instead, it arises from exposures that are moderately associated with distress but occur often enough to impact many people.

At the individual level, the highest risk of psychological distress stems from severe SACEs, such as being “hit hard enough to leave bruises” or being “hurt for not doing something sexual,” with risk increasing as exposure frequency rises. In contrast, at the population level, the greatest impact on psychological distress comes from SACEs that, while less severe on an individual basis (e.g., being “called names by family” occurring “sometimes”), are prevalent enough to affect a significant portion of the population.

### Implications

Our findings offer important insights for both research and public health. Firstly, the SACEs with the most significant impact at the individual level — those crucial to address in treatment settings — are not necessarily the same as those contributing most to the overall burden of psychological distress at the population level. From a public health perspective, it is essential to focus not only on severe SACEs with observable signs, such as physical abuse, but also on less visible forms like emotional maltreatment [15, 17, 45, 46]. While these less visible SACEs, at a low or moderate exposure frequency, may be less severe for each individual, their high prevalence makes them significant targets for prevention and public health initiatives. However, such efforts should not focus solely on the overall prevalence rate of these SACEs, but also aim to reduce the frequency of such experiences to a less harmful level and support individuals in coping with their impact. From a population-level perspective, broad prevention strategies also targeting family environments with moderate dysfunctions mainly in the emotional domain are just as important as those aimed at families with severe dysfunctions involving physical or sexual maltreatment.

Secondly, building composite scores for ACE that assign equal weight to each exposure frequency (e.g., scoring “rarely” as 2 and “very often” as 5) may be problematic. This approach implies that all exposure frequencies of all SACEs have equal associations with outcomes [20] which differs from the results of our study. More research across diverse populations, settings, and life stages is needed to better understand the threshold at which a normal experience becomes an adverse one. It is also crucial to examine how other risk and protective factors influence this threshold on an individual level.

While our results highlight the limitations of composite scores and dimensional approaches [20], we do not aim to discourage their use but rather recommend complementing them with approaches that consider the specific associations of individual SACEs and their exposure frequencies.

### Limitations

In line with the primary aims of our study and to ensure the interpretability of our results, we reported only bivariate associations between the frequency of each SACE and psychological distress. Our single-risk approach does not account for the co-occurrence of different SACEs and their combined effects on psychological distress [20]. Furthermore, this study did not account for the impact of recent life events and resilience factors which may affect the association between SACEs and psychological distress. Our future research will employ comprehensive longitudinal models to explore the mechanisms linking ACE patterns to psychosocial functioning, while accounting for potential covariates, life events and resilience factors.

Then, despite the large list-based random sample of emerging adults in German-speaking Switzerland, the relatively low response rate and an overrepresentation of women limit the generalizability of the results. Furthermore, despite our large sample size, cell sizes for rarely reported SACEs at higher exposure frequencies were small, warranting caution when interpreting these associations. Finally, we assessed ACE as retrospective self-reports which were not corroborated with agency-, parent- or teacher-reported data. Therefore, the results may be subject to recall and reporting bias [47]. A further limitation concerns the objectivity of the CTQ frequency ratings themselves. While the response category “never” can be considered relatively unambiguous, the remaining categories (e.g., “rarely,” “sometimes,” “often,” “very often”) rely on subjective interpretation. These terms may vary both between individuals and within the same individual over time, potentially introducing measurement variability. Such subjective judgments may be influenced by personal interpretation, recall processes, or the respondent’s current psychological state. This limitation applies to many retrospective self-report instruments assessing childhood maltreatment; and, in line with LaNoue & Hass [48], the responses on self-report questionnaires should be considered as “a mix of perceptions, recollections, and meaning-making around childhood experiences”.

## Conclusion

This study highlights the profound impact of adverse childhood experiences on psychological distress in emerging adults, showing that this impact varies based on the type and frequency of adversity experienced. Additionally, our research underscores the value of single adversity approaches as a complement to dimensional approaches in ACE research considering both individual and population-level effects. These complementary perspectives are crucial for informing treatment, prevention and public health initiatives, both at the individual and population levels.

Figures captions and tables.

## Supplementary Information

Supplementary Material 1.

## Data Availability

The data that support the findings of this study are available from the corresponding author, [JB], upon reasonable request.
