# Supplementary material for: Exposure frequencies of single adverse childhood experiences and their association with psychological distress: evidence from a cohort study among emerging Swiss adults
Source: BMC Public Health. 2025 Dec 24;26:482. doi: 10.1186/s12889-025-25999-6 (PMC12874928; doi:10.1186/s12889-025-25999-6)
Supplement: Supplementary file 1 — Supplementary Material 1. [file 12889_2025_25999_MOESM1_ESM.pdf]

Supplementary Table 1. Prevalence of exposure frequencies for each SACE by gender

		never (1)	rarely (2)	some-times (3)	often (4)	very often (5)	mean	SD	chi-squared	p-value
**Emotional neglect**										
Made to feel important (R)	women	48.21	30.76	13.47	5.34	2.23	1.83	1.00		
	men	44.35	31.85	16.27	6.16	1.37	1.88	0.98	5.589	0.232
	non-binary, other	22.86	28.57	28.57	14.29	5.71	2.51	1.17	16.917	**0.002**
	total	46.53	31.06	14.62	5.76	2.03	1.86	1.00		
Felt loved (R)	women	54.19	28.01	10.38	6.15	1.28	1.72	0.96		
	men	53.42	27.57	12.50	5.31	1.20	1.73	0.95	2.193	0.700
	non-binary, other	31.43	34.29	22.86	5.71	5.71	2.20	1.13	13.527	**0.009**
	total	53.53	27.99	11.27	5.88	1.34	1.74	0.97		
Was looked out for (R)	women	42.49	35.78	13.42	6.63	1.68	1.89	0.98		
	men	47.69	36.75	9.57	4.79	1.20	1.75	0.90	10.228	**0.037**
	non-binary, other	17.14	34.29	28.57	14.29	5.71	2.57	1.12	16.932	**0.002**
	total	43.64	36.06	12.50	6.20	1.60	1.86	0.97		
Family felt close (R)	women	36.31	36.07	15.48	9.18	2.95	2.06	1.07		
	men	40.75	36.82	14.38	5.99	2.05	1.92	0.99	8.613	0.072
	non-binary, other	17.14	25.71	25.71	20.00	11.43	2.83	1.27	18.672	**0.001**
	total	37.34	36.11	15.33	8.39	2.83	2.03	1.06		
Family was source of strength (R)	women	48.20	28.65	12.61	7.74	2.79	1.88	1.08		
	men	50.94	27.79	12.18	6.52	2.57	1.82	1.05	1.653	0.799
	non-binary, other	14.29	25.71	37.14	14.29	8.57	2.77	1.14	29.518	**0.000**
	total	48.42	28.33	12.93	7.48	2.83	1.88	1.07		

		never (1)	rarely (2)	sometimes (3)	often (4)	very often (5)	mean	SD	chi-squared	p-value
**Physical neglect**										
Not enough to eat	women	95.30	3.04	0.96	0.70		1.07	0.36		
	men	94.04	3.85	1.54	0.58		1.09	0.38	1.903	0.593
	non-binary, other	87.10	6.45	6.45	0.00		1.19	0.54	17.609	**0.001**
	total	94.77	3.35	1.23	0.65		1.08	0.37		
Got taken care of (R)	women	69.01	22.81	5.62	2.56		1.42	0.71		
	men	68.77	23.16	5.61	2.46		1.42	0.71	0.041	0.998
	non-binary, other	53.13	18.75	15.63	12.50		1.88	1.10	17.609	**0.001**
	total	68.65	22.85	5.79	2.70		1.43	0.72		
Parents were drunk or high	women	93.94	2.63	1.83	0.72	0.88	1.12	0.54		
	men	93.50	1.88	2.39	1.20	1.03	1.14	0.61	2.715	0.607
	non-binary, other	71.43	14.29	2.86	5.71	5.71	1.60	1.17	35.588	**0.000**
	total	93.38	2.61	2.03	0.96	1.01	1.14	0.58		
Wore dirty clothes	women	94.96	4.16	0.64	0.08	0.16	1.06	0.31		
	men	91.11	5.13	2.39	0.85	0.51	1.15	0.53	20.785	**0.000**
	non-binary, other	88.57	5.71	2.86	2.86	0.00	1.20	0.63	19.641	**0.001**
	total	93.63	4.49	1.23	0.37	0.27	1.09	0.40		
Got taken to doctor (R)	women	84.68	12.04	2.54	0.74		1.19	0.50		
	men	87.15	9.72	2.26	0.87		1.17	0.49	2.352	0.503
	non-binary, other	73.53	17.65	8.82	0.00		1.35	0.65	6.414	0.093
	total	85.25	11.41	2.57	0.76		1.19	0.50		

		never (1)	rarely (2)	sometimes (3)	often (4)	very often (5)	mean	SD	chi-squared	p-value
**Emotional abuse**										
Called names by family	women	47.33	27.45	15.40	6.38	3.43	1.91	1.53		
	men	50.17	28.25	13.36	5.65	2.57	1.82	1.32	3.181	0.528
	non-binary, other	28.57	20.00	22.86	11.43	17.14	2.69	2.29	22.618	**0.000**
	total	47.86	27.56	14.90	6.25	3.42	1.90	1.48		
Parents wished was never born	women	70.74	14.87	7.75	4.16	2.48	1.09	0.98		
	men	81.71	10.09	4.62	2.05	1.54	1.03	0.79	25.786	**0.000**
	non-binary, other	48.57	20.00	2.86	11.43	17.14	1.45	1.58	33.639	**0.000**
	total	73.76	13.47	6.68	3.63	2.46	1.08	0.95		
Family said hurtful things	women	36.88	32.96	18.00	7.36	4.80	2.10	1.13		
	men	48.89	32.31	11.45	4.79	2.56	1.80	0.99	33.814	**0.000**
	non-binary, other	34.29	20.00	14.29	25.71	5.71	2.49	1.36	16.73	**0.002**
	total	40.59	32.51	15.88	6.90	4.12	2.01	1.10		
Felt hated by family	women	67.95	15.11	8.79	4.96	3.20	1.60	1.05		
	men	74.32	14.38	5.65	2.57	3.08	1.46	0.94	12.919	**0.012**
	non-binary, other	54.29	14.29	8.57	14.29	8.57	2.09	1.42	9.586	**0.048**
	total	69.68	14.87	7.81	4.39	3.26	1.57	1.03		
Was emotionally abused	women	73.28	10.88	7.92	4.00	3.92	1.54	1.06		
	men	84.93	7.19	3.25	1.71	2.91	1.30	0.85	33.563	**0.000**
	non-binary, other	37.14	25.71	14.29	8.57	14.29	2.37	1.44	24.925	**0.000**
	total	76.24	10.01	6.58	3.37	3.80	1.48	1.02		

		never (1)	rarely (2)	sometimes (3)	often (4)	very often (5)	mean	SD	chi-squared	p-value
**Physical abuse**										
Hit hard enough to leave bruises	women	88.03	7.58	3.11	1.12	0.16	1.18	0.54		
	men	86.84	8.03	3.59	1.03	0.51	1.20	0.60	2.308	0.679
	non-binary, other	80.00	14.29	0.00	5.71	0.00	1.31	0.76	9.158	0.057
	total	87.51	7.85	3.20	1.17	0.27	1.19	0.57		
Hit hard enough to see doctor	women	98.40	1.04	0.48	0.08	0.00	1.02	0.19		
	men	98.29	1.20	0.17	0.17	0.17	1.03	0.25	3.534	0.473
	non-binary, other	100.00	0.00	0.00	0.00	0.00	1.00	0.00	0.568	0.904
	total	98.40	1.07	0.37	0.11	0.05	1.02	0.21		
Punished with hard objects	women	89.06	6.31	3.27	1.04	0.32	1.17	0.56		
	men	87.33	5.99	5.31	0.51	0.86	1.22	0.64	7.996	0.092
	non-binary, other	91.43	2.86	0.00	2.86	2.86	1.23	0.84	8.5	0.075
	total	88.56	6.15	3.85	0.91	0.53	1.19	0.59		
Was physically abused	women	91.04	5.20	2.08	1.04	0.64	1.15	0.56		
	men	90.58	5.65	1.71	1.54	0.51	1.16	0.57	1.374	0.849
	non-binary, other	80.00	11.43	2.86	0.00	5.71	1.40	1.01	14.644	**0.005**
	total	90.69	5.46	1.98	1.18	0.70	1.16	0.57		
Hit badly enough to be noticed	women	97.52	1.84	0.48	0.08	0.08	1.03	0.24		
	men	96.91	1.37	1.20	0.17	0.34	1.06	0.36	5.438	0.245
	non-binary, other	94.29	2.86	2.86	0.00	0.00	1.09	0.37	3.822	0.431
	total	97.27	1.71	0.75	0.11	0.16	1.04	0.28		

		never (1)	rarely (2)	sometimes (3)	often (4)	very often (5)	mean	SD	chi-squared	p-value
**Sexual abuse**										
Was touched sexually	women	84.81	8.07	4.64	1.76	0.72	1.25	0.69		
	men	96.06	1.88	1.54	0.17	0.34	1.07	0.38	50.088	**0.000**
	non-binary, other	58.82	8.82	17.65	11.76	2.94	1.91	1.24	32.41	**0.000**
	total	87.85	6.15	3.91	1.44	0.64	1.21	0.64		
Hurt if didn't do something sexual	women	96.56	1.28	1.44	0.24	0.48	1.07	0.41		
	men	99.14	0.51	0.17	0.17	0.00	1.01	0.17	11.59	**0.021**
	non-binary, other	88.24	5.88	5.88	0.00	0.00	1.18	0.52	9.689	**0.046**
	total	97.22	1.12	1.12	0.21	0.32	1.05	0.35		
Made to do sexual things	women	92.01	3.76	2.64	0.88	0.72	1.15	0.56		
	men	97.60	1.71	0.34	0.34	0.00	1.03	0.25	23.507	**0.000**
	non-binary, other	70.59	14.71	11.76	2.94	0.00	1.47	0.83	22.793	**0.000**
	total	93.37	3.32	2.09	0.75	0.48	1.12	0.50		
Was molested	women	82.56	9.84	5.12	1.36	1.12	1.29	0.72		
	men	97.09	1.71	0.86	0.17	0.17	1.05	0.30	75.241	**0.000**
	non-binary, other	58.82	11.76	20.59	8.82	0.00	1.79	1.07	28.94	**0.000**
	total	86.67	7.33	4.07	1.12	0.80	1.22	0.65		
Was sexually abused	women	89.34	6.09	2.64	0.88	1.04	1.18	0.62		
	men	97.77	1.20	0.69	0.34	0.00	1.04	0.26	39.255	**0.000**
	non-binary, other	67.65	14.71	8.82	8.82	0.00	1.59	0.99	29.617	**0.000**
	total	91.58	4.72	2.14	0.86	0.70	1.14	0.55		

		never (1)	rarely (2)	sometimes (3)	often (4)	very often (5)	mean	SD	chi-squared	p-value
**MACE Witnessing violence**										
Parents fighting	women	22.33	39.07	17.07	11.40	10.13	2.48	1.24		
	men	28.08	40.75	19.01	5.82	6.34	2.22	1.11	25.904	**0.000**
	non-binary, other	8.57	28.57	20.00	25.71	17.14	3.14	1.26	11.612	**0.020**
	total	23.87	39.40	17.73	9.93	9.08	2.41	1.21		
Violence between parents	women	77.27	12.04	6.22	2.07	2.39	1.40	0.88		
	men	80.31	13.01	3.94	1.71	1.03	1.30	0.72	8.604	0.072
	non-binary, other	62.86	17.14	14.29	2.86	2.86	1.66	1.03	5.218	0.266
	total	77.95	12.44	5.66	1.98	1.98	1.38	0.84		
Parent got injured	women	93.31	3.27	1.91	0.56	0.96	1.13	0.54		
	men	95.03	3.26	0.86	0.34	0.51	1.08	0.42	4.258	0.372
	non-binary, other	85.71	5.71	8.57	0.00	0.00	1.23	0.60	8.567	0.073
	total	93.70	3.31	1.71	0.48	0.80	1.11	0.51		
Violence towards sibling	women	74.50	15.11	7.11	2.24	1.04	1.40	0.80		
	men	75.68	15.24	6.34	2.23	0.51	1.37	0.75	1.692	0.792
	non-binary, other	65.71	11.43	11.43	8.57	2.86	1.71	1.15	8.236	0.083
	total	74.71	15.08	6.95	2.35	0.91	1.40	0.79		
Sibling got injured	women	94.33	3.67	1.04	0.80	0.16	1.09	0.41		
	men	95.20	2.92	1.03	0.51	0.34	1.08	0.41	1.759	0.780
	non-binary, other	94.29	2.86	2.86	0.00	0.00	1.09	0.37	1.434	0.838
	total	94.60	3.42	1.07	0.69	0.21	1.08	0.41		

		never (1)	rarely (2)	sometimes (3)	often (4)	very often (5)	mean	SD	chi-squared	p-value
**MACE Peers verbal**							2.44	1.24		
Called names by peers	women	27.72	28.83	22.92	12.38	8.15	2.35	1.10		
	men	24.62	36.07	24.62	9.57	5.13	3.66	1.21	16.414	**0.003**
	non-binary, other	5.71	14.29	17.14	34.29	28.57	2.44	1.21	38.024	**0.000**
	total	26.34	30.82	23.34	11.91	7.59	2.50	1.26		
Verbal abuse by peers	women	26.70	28.06	22.86	13.51	8.87	2.27	1.17		
	men	30.26	34.02	20.00	9.74	5.98	3.51	1.22	16.804	**0.002**
	non-binary, other	2.86	22.86	22.86	22.86	28.57	2.45	1.24	23.962	**0.000**
	total	27.37	29.82	21.97	12.51	8.34	2.45	1.25		
Excluded by peers	women	27.50	29.50	22.78	11.35	8.87	2.25	1.19		
	men	32.36	33.22	17.47	10.96	5.99	3.69	1.28	14.582	**0.006**
	non-binary, other	5.71	14.29	22.86	20.00	37.14	2.41	1.25	39.132	**0.000**
	total	28.61	30.37	21.12	11.39	8.50				
**MACE Peers physical**										
Hit by peers	women	76.22	15.93	5.20	1.04	1.60	1.36	0.77		
	men	55.75	29.67	9.43	2.74	2.40	1.66	0.93	80.381	**0.000**
	non-binary, other	37.14	31.43	17.14	14.29	0.00	2.09	1.07	63.857	**0.000**
	total	69.09	20.51	6.75	1.82	1.82	1.47	0.84		
Injured by peers	women	93.66	4.09	1.20	0.48	0.56	1.10	0.46		
	men	85.98	8.55	4.10	0.68	0.68	1.22	0.61	33.052	**0.000**
	non-binary, other	71.43	25.71	2.86	0.00	0.00	1.31	0.53	36.936	**0.000**
	total	90.84	5.89	2.14	0.54	0.59	1.14	0.52		
Sexual violence by peers	women	95.20	2.96	1.36	0.32	0.16	1.07	0.37		
	men	97.95	1.37	0.34	0.17	0.17	1.03	0.26	8.734	0.068
	non-binary, other	71.43	11.43	17.14	0.00	0.00	1.46	0.78	57.183	**0.000**
	total	95.61	2.62	1.34	0.27	0.16	1.07	0.35		

Note: bold coefficients are statistically significant at a p-value ≤ 0.05.

Supplementary Table 2. Prevalence, severity levels and means for CTQ ACE scales by gender

		CTQ scale severity levels in %					scale mean			
		none to minimal	slight to moderate	moderate to severe	severe to extreme	total: at least slight to moderate	mean	SD	t-test	p-value
Emotional neglect	women	60.85	25.20	6.94	7.02	39.15	9.39	4.43		
	men	65.47	21.88	8.03	4.62	34.53	9.11	4.13	1.317	0.176
	non-binary, other	31.43	31.43	14.29	22.86	68.57	12.89	5.06	-4.595	**0.001**
	total	61.58	24.32	7.42	6.68	38.42	9.36	4.38		
Physical neglect	women	89.06	8.07	2.08	0.80	10.94	5.86	1.51		
	men	89.23	5.30	3.59	1.88	10.77	5.96	1.88	-1.137	0.21269
	non-binary, other	68.57	14.29	11.43	5.71	31.43	7.25	3.18	-2.588	**0.044**
	total	88.65	7.37	2.71	1.27	11.35	5.92	1.69		
Emotional abuse	women	66.40	17.30	5.90	10.50	33.70	8.69	4.40		
	men	77.60	12.30	5.30	4.80	22.40	7.70	3.74	4.968	**0.001**
	non-binary, other	40.00	20.00	8.60	31.40	60.00	11.91	6.11	-3.100	**0.005**
	total	69.23	15.86	5.78	9.12	30.77	8.44	4.29		
Physical abuse	women	93.10	3.00	2.20	1.60	6.80	5.56	1.63		
	men	92.60	3.10	2.70	1.50	7.30	5.66	1.97	-1.224	0.232
	non-binary, other	94.30	0.00	0.00	5.70	5.70	6.03	2.83	-0.979	0.353
	total	92.94	3.02	2.33	1.70	7.06	5.60	1.77		
Sexual abuse	women	78.00	10.60	7.60	3.80	22.00	5.94	2.61		
	men	94.30	2.90	2.40	0.30	5.60	5.20	1.10	8.514	**0.001**
	non-binary, other	55.90	2.90	29.40	11.80	44.10	7.94	4.08	-2.848	**0.0026**
	total	82.53	8.13	6.43	2.92	17.47	5.74	2.33		

		CTQ scale severity levels in %					scale mean			
		none to minimal	slight to moderate	moderate to severe	severe to extreme	total: at least slight to moderate	mean	SD	t-test	p-value
MACE Witnessing violence	women						7.50	3.00		
	men						7.04	2.56	3.353	**0.002**
	non-binary, other						8.83	3.10	-2.580	**0.043**
	total						7.38	2.89		
Peers verbal	women						7.39	3.44		
	men						6.87	3.15	3.213	**0.004**
	non-binary, other						10.86	3.26	-5.896	**0.000**
	total						7.29	3.39		
Peers physical	women						3.54	1.29		
	men						3.91	1.50	-5.184	**0.000**
	non-binary, other						4.86	1.80	-4.308	**0.002**
	total						3.68	1.39		

Note: bold coefficients are statistically significant at a p-value ≤ 0.05. Severity cut-offs for CTQ scales were used as defined in Häuser et al. (2011). Prevalence rates for MACE subscales are not shown as they are no validated cut-offs. p-values have been bootstrapped with 1000 bootstraps each.

Supplementary Table 3. Prevalence of parental psychopathology by gender

		none	father only	mother only	both parents	chi-squared	p-value
Alcohol problem	women	91.09	6.13	1.75	1.03		
	men	93.02	4.60	1.53	0.85	2.062	0.560
	non-binary, other	77.14	17.14	2.86	2.86	8.443	**0.038**
	total	91.43	5.85	1.70	1.01		
Drug problem	women	97.85	1.83	0.24	0.08		
	men	98.13	1.19	0.51	0.17	2.219	0.528
	non-binary, other	88.57	8.57	2.86	0.00	15.56	**0.001**
	total	97.76	1.76	0.37	0.11		
Depression	women	75.89	5.89	15.35	2.86		
	men	82.96	4.43	10.56	2.04	11.804	**0.008**
	non-binary, other	54.29	8.57	17.14	20.00	32.638	**0.000**
	total	77.70	5.48	13.89	2.93		
Delusions/hallucinations	women	98.49	0.56	0.95	0.00		
	men	97.61	0.34	2.04	0.00	4.064	0.131
	non-binary, other	97.14	2.86	0.00	0.00	3.25	0.197
	total	98.19	0.53	1.28	0.00		
Suicide attempt	women	97.45	0.72	1.75	0.08		
	men	97.96	0.68	1.36	0.00	0.853	0.837
	non-binary, other	97.14	0.00	2.86	0.00	0.513	0.916
	total	97.61	0.69	1.65	0.05		
Other psychological problems	women	82.90	5.57	8.83	2.70		
	men	87.73	3.92	6.64	1.70	7.304	0.063
	non-binary, other	60.00	5.71	14.29	20.00	35.397	**0.000**
	total	83.98	5.06	8.25	2.71		
Aggressive behaviour	women	85.36	10.34	2.15	2.15		
	men	85.86	8.01	3.75	2.39	6.241	0.100
	non-binary, other	65.71	28.57	2.86	2.86	12.15	**0.007**
	total	85.15	9.95	2.66	2.24		

Note: bold coefficients are statistically significant at a p-value ≤ 0.05.

Supplementary Table 4. Associations between specific SACE’s exposure frequencies and psychological distress (BSI score non-standardized)

	Adverse childhood experience	marginal means (z-standardized BSI scores)					partial eta squared (% of variance explained)				
		never	very rarely	sometimes	often	very often	total item	very rarely	sometimes	often	very often
Emotional neglect	Made to feel important (R)	13.98	17.28	21.93	26.71	32.08	13.8	1.8	6.0	6.9	5.5
	Felt loved (R)	14.22	18.26	22.94	29.00	34.92	16.4	2.7	6.2	9.7	5.0
	Was looked out for (R)	13.77	17.14	22.42	27.32	32.34	14.3	2.0	6.2	8.3	4.6
	Family felt close (R)	13.52	16.74	19.24	27.49	29.18	14.4	1.7	3.1	10.8	5.4
	family was source of strength (R)	13.06	17.03	21.82	26.29	33.77	19.2	2.7	7.0	9.8	10.0
Physical neglect	Not enough to eat	18.10	25.54	30.46	31.36		3.6	1.4	1.5	0.9	
	Got taken care of (R)	14.82	19.92	26.01	30.96		12.1	3.9	5.7	5.8	
	Parents were drunk or high	16.82	25.61	28.37	25.21	28.34	4.9	1.6	2.2	0.6	1.1
	Wore dirty clothes	17.65	21.06	26.51	28.35	29.30	1.7	0.4	0.8	0.3	0.3
	Got taken to doctor (R)	17.14	20.21	22.82	28.33		2.0	0.8	0.7	0.8	
Emotional abuse	Called names by family	13.78	16.85	22.53	25.45	29.30	14.9	1.5	7.4	6.5	6.6
	Parents wished was never born	14.21	20.66	24.73	29.60	36.29	20.3	4.5	6.3	7.4	10.2
	Family said hurtful things	14.11	16.63	22.28	25.24	31.44	15.9	1.1	6.6	6.3	9.5
	Felt hated by family	15.10	19.25	23.93	27.28	33.11	15.5	1.9	4.8	5.4	8.6
	Was emotionally abused	14.95	19.82	22.92	26.77	32.29	14.1	1.9	3.3	3.9	9.0
Physical abuse	Hit hard enough to leave bruises	16.91	23.85	25.74	28.52	38.40	6.3	2.8	2.0	1.3	1.0
	Hit hard enough to see doctor	18.08	24.04	34.23	13.63	27.96	1.1	0.3	0.8	0.0	0.0
	Punished with hard objects	17.44	20.85	24.78	26.74	32.45	3.4	0.5	1.6	0.6	1.0
	Was physically abused	16.84	24.79	26.61	30.02	31.82	6.4	2.7	1.5	1.7	1.3
	Hit badly enough to be noticed	17.61	31.24	35.47	15.14	19.70	4.3	2.5	1.9	0.0	0.0

Commented [SM1]: Evlt doch noch mit effect size farben einfach cohen’s d * SD multiplizieren

Sexual abuse	Was touched sexually	16.25	21.63	24.63	28.60	26.05	4.9	1.3	2.1	1.7	0.5
	Hurt if didn't do something sexual	17.08	32.82	33.53	30.99	25.51	4.9	2.2	2.4	0.3	0.2
	Made to do sexual things	16.74	22.65	29.18	29.30	22.62	4.2	0.9	2.5	1.0	0.1
	Was molested	16.16	20.64	27.23	28.14	26.60	5.9	1.1	3.7	1.3	0.7
	Was sexually abused	16.49	23.31	27.68	30.15	28.17	5.2	1.7	2.1	1.3	0.8
Witnessing violence	Parents fighting	14.62	16.06	18.38	21.86	25.71	8.6	0.3	1.2	3.0	6.5
	Violence between parents	16.97	18.91	22.47	23.34	32.61	5.4	0.3	1.3	0.7	3.8
	Parent got injured	17.57	21.69	25.06	32.46	34.46	3.7	0.4	0.8	0.9	1.8
	Violence towards sibling	16.84	19.05	20.47	26.60	37.79	5.4	0.5	0.7	1.8	3.2
	Sibling got injured	17.71	23.96	27.69	29.41	40.85	3.4	1.0	0.9	0.8	0.9
Peers verbal	Called names by peers	12.84	14.39	17.26	21.29	29.85	16.4	0.3	2.2	5.2	13.7
	Verbal abuse by peers	13.35	14.94	16.82	22.32	28.81	15.5	0.3	1.3	6.0	12.3
	Excluded by peers	13.29	14.85	16.23	21.67	28.57	14.7	0.3	1.0	5.0	12.1
Peers physical	Hit by peers	15.29	18.35	25.80	27.70	30.38	9.9	1.2	5.5	2.3	3.4
	Injured by peers	16.91	23.13	28.53	28.77	37.27	6.0	1.7	2.3	0.6	2.0
	Sexual violence by peers	16.72	24.39	32.55	21.62	41.85	4.4	1.2	2.6	0.1	0.8
		No parent	One parent	Both parents			No parent	One parent	Both parents		
Parental psychopathology	Substance use problems	16.91	24.65	28.14			4.6	3.5	1.4		
	Psychological problems	15.69	20.71	25.64			6.8	3.5	4.5		
	Aggressive behavior	16.26	21.85	29.22			5.8	2.9	3.5		

Note: All statistics are independent of each other and adjusted for age and gender.
